# Scar endometrioma following obstetric surgical incisions: retrospective study on 33 cases and review of the literature

**DOI:** 10.1590/S1516-31802009000500005

**Published:** 2010-02-03

**Authors:** Guilherme Karam Corrêa Leite, Luis Fernando Pina de Carvalho, Henri Korkes, Thiago Falbo Guazzelli, Grecy Kenj, Arildo de Toledo Viana

**Affiliations:** I Resident in gynecology and obstetrics, Hospital Municipal Maternidade-Escola Dr. Mário de Moraes Altenfelder Silva, São Paulo, São Paulo, Brazil.; II MD. Attending physician in gynecology and obstetrics, Hospital Municipal Maternidade-Escola Dr. Mário de Moraes Altenfelder Silva, São Paulo, São Paulo, Brazil.; III MD, PhD. Associate full professor and coordinator of the Discipline of Surgical Technique, Faculdade de Ciências Médicas da Santa Casa de São Paulo (FCMSCSP), São Paulo, and scientific-technical coordinator of Clinical Surgery, Hospital Municipal Maternidade-Escola Dr. Mário de Moraes Altenfelder Silva, São Paulo, São Paulo, Brazil.

**Keywords:** Endometriosis, Cicatrix, Cesarean section, Postoperative complications, Treatment outcome, Endometriose, Cicatriz, Cesárea, Complicações pós-operatórias, Resultado de tratamento

## Abstract

**CONTEXT AND OBJECTIVE:**

The incidence of scar endometrioma ranges from 0.03 to 3.5%. Certain factors relating to knowledge of the clinical history of the disease make correct diagnosis and treatment difficult. The aim here was to identify the clinical pattern of the disease and show surgical results. The literature on this topic was reviewed.

**DESIGN AND SETTING:**

Retrospective descriptive study at Hospital Municipal Maternidade - Escola Dr. Mário de Moraes Altenfelder Silva.

METHODS: Data from the medical records of patients with preoperative diagnoses of scar endometrioma who underwent operations between 2001 and 2007 were surveyed and reviewed. The postoperative diagnosis came from histopathological analysis. The main information surveyed was age, obstetric antecedents, symptoms, tumor location, size and palpation, duration of complaint, diagnosis and treatment. All patients underwent tumor excision with a safety margin.

**RESULTS:**

There were 33 patients, of mean age 30.1 ± 5.0 years (range: 18-41 years). The total incidence was 0.11%: 0.29% in cesarean sections and 0.01% in vaginal deliveries. Twenty-nine tumors (87.9%) were located in cesarean scars, two (6.0%) in episiotomy scars and two (6.0%) in the umbilical region. The main symptom was localized cyclical pain (66.7%), of mean duration 30.5 months (± 23). Surgical treatment was successful in all cases.

**CONCLUSION:**

This is an uncommon disease. The most important diagnostic characteristic is coincidence of painful symptoms with menstruation. Patients undergoing cesarean section are at greatest risk: relative risk of 27.37 (P < 0.01). The surgical treatment of choice is excision of the endometrioma with a safety margin.

## RESUMO

**CONTEXTO E OBJETIVO:**

A incidência de endometrioma de cicatriz varia de 0,03 a 3,5%. Alguns fatores relacionados ao conhecimento da história clínica da doença dificultam o diagnóstico e o tratamento corretos. O objetivo é traçar o padrão clínico da doença e avaliar resultados cirúrgicos. Foi revisada a literatura sobre o assunto.

**TIPO DE ESTUDO E LOCAL:**

Estudo descritivo e retrospectivo realizado no Hospital Municipal Maternidade - Escola Dr. Mário de Moraes Altenfelder Silva.

**MÉTODOS:**

Foi realizado levantamento e revisão de dados dos prontuários médicos das pacientes com diagnóstico pré-operatório de endometrioma de cicatriz, operadas entre 2001 e 2007. O diagnóstico pós-operatório foi feito por exame histopatológico. As principais informações levantadas foram: idade, antecedentes obstétricos, sintomatologia, localização, tamanho e palpação do tumor, duração da queixa, diagnóstico, tratamento. Todas as pacientes foram submetidas a exerese da massa tumoral com margem de segurança.

**RESULTADOS:**

Foram encontrados 33 pacientes com média de idade 30,1 (± 5,0), variando de 18 a 41 anos. A incidência total foi de 0,11%, nas cesarianas foi de 0,29% e nos partos vaginais, 0,01%. Localização do tumor: 29 casos em cicatriz de cesária (87,9%), dois em região umbilical (6,0%) e dois em cicatriz de episiotomia (6,0%). A principal sintomatologia foi dor cíclica localizada (66,7%), com duração média de 30,5 meses (± 23). O tratamento cirúrgico foi realizado com sucesso em todas as pacientes.

**CONCLUSÃO:**

Trata-se de uma doença incomum. O dado de maior importância diagnóstica foi: coincidência da sintomatologia dolorosa com a menstruação. Pacientes submetidas a cesariana têm maior risco (risco relativo = 27,37 e P < 0,01). O tratamento cirúrgico de escolha é exerese do endometrioma com margem de segurança.

## INTRODUCTION

The presence of ectopic endometrial tissue, called endometriosis, is more commonly found within the female pelvic cavity, attacking the ovaries, the rectovaginal pouch and the peritoneum of the genital floor. Less frequently, it can occur in extrapelvic sites, especially in abdominal surgery scar areas following hysterectomy and cesarean section, and in the perineum following vaginal deliveries with episiotomy.^1^ Other remote sites have been described, such as the extremities, central nervous system, lungs, pleurae, liver, umbilicus, pericardium, urinary tract and intestines; however, these are rare events.^2^

Reports in the literature state that endometriosis may be present in surgical scars following laparotomy, laparoscopy and diagnostic obstetric procedures such as amniocentesis puncture.^3^ Furthermore, this disease is also related to surgery performed by general surgeons, such as appendectomy, groin and umbilical hernia corrections.^4^^,^^5^ However, most of the cases reported have occurred following obstetric procedures that exposed the endometrial tissue, especially in cases of cesarean section.^6^^,^^7^^,^^8^

**Figure 1.**
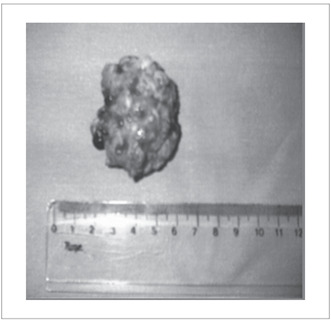
Endometrioma after resection. Note tumor formation consisting of whitish fibrous tissue and chocolate-like area on the surface.

The term scar endometrioma is used for well-marked tumoral lesions, such as non-neoplastic granuloma or tumor. It is formed by whitish fibrous tissue, with thick chocolate-like colored liquid areas, and is located anywhere in the surgical scar^7^ (Figure 1). Not all scar endometriosis is characterized by endometrioma, and this makes diagnosis difficult when there are no palpable nodules.^2^

The incidence of endometrioma in the worldwide literature ranges from 0.03 to 3.5%.^8^^,^^9^^,^^10^ Its frequency in episiotomy scars is much smaller than in abdominal wall scars.^6^ The theory of iatrogenic implantation is the one most accepted by several authors, and other theories are secondary to this, in order to explain the physiopathology.^11^^,^^12^ Certain factors relating the knowledge of the clinical background of the disease make correct diagnosis difficult, especially among general surgeons, who most commonly make mistakes during diagnosis.^13^ There is no need for advanced propaedeutics, and the diagnosis may be made solely based on anamnesis and physical examination, aided by ultrasound in some special cases. However, the diagnosis must always be defined through anatomopathological examination. The treatment is basically surgical, and the use of medications that have already been proven for treating pelvic endometriosis has been described for controlling scar endometrioma.^14^^,^^15^

## OBJECTIVE

The aim of this study was to identify risk factors and show the clinical patterns and other forms of presentation of scar endometrioma, through publishing the results from our experience of surgical management of such lesions. Furthermore, we proposed to add data from the recent literature, to produce an up-to-date review on this subject.

## METHODS

**Figure 2.**
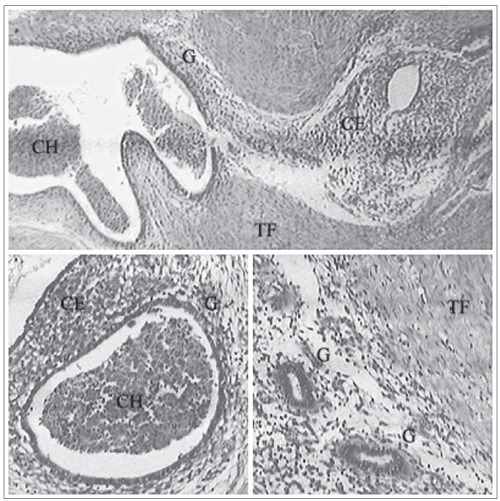
Immunohistochemistry (hematoxylin-eosin x 100). Fibromuscular tissue (TF) with glandular structures marked out by lines of cubic cells (G), girdles for stromal endometrial cells with reduced cytoplasm (CE) and circumscribed hematic cells and debris (CH).

This was a descriptive, observational, retrospective cohort study performed at the Hospital Municipal Maternidade-Escola de Vila Nova Cachoeirinha Dr. Mário de Moraes Altenfelder Silva (HMMEVNC). It consisted of surveying and reviewing data from the medical records of patients diagnosed with surgical scar endometrioma prior to their surgery. These operations took place between August 2001 and December 2007. The postsurgical diagnoses was performed using histopathological analysis, in which the criterion was the presence of endometrial glands and/or stromal cells in the connective tissue that was analyzed, i.e. in subcutaneous, fibrous or muscle connective tissue (Figure 2).

The main information surveyed was age, obstetric antecedents, symptoms, tumor location, size and palpation, recurrent lesions, duration of the complaint, diagnosis, treatment and asymptomatic window (the time interval between the obstetric procedure and the onset of symptoms). All the patients underwent surgical removal of the tumor with a safety margin, and the definitive diagnosis was confirmed by the pathological anatomical examination.

This research project was approved by the Research Ethics Committee of HMMEVNC. The data were cross-referenced and processed using the Epi-Info 3.4.3 statistical software, and Student’s test was used to estimate the variables. The incidence estimate was based in the absolute number of obstetric surgical procedures performed in our institution over the period surveyed, comparing the different types of delivery. The results were taken to be significant when P < 0.05. We reviewed the literature using the Medical Literature Analysis and Retrieval System Online (Medline), Scientific Electronic Library Online (SciELO) and Literatura Latino-Americana e do Caribe em Ciências da Saúde (Lilacs) databases, which mostly made case reports available (Table 1).

**Table 1.** Database search strategies and outcomes

Database	Search strategies	Outcomes
Medline	Endometriosis [MeSH] AND Cesarean section [MeSH] AND Cicatrix [MeSH]	66 case reports 36 case series 4 narrative reviews
Lilacs (Literatura Latino-Americana e do Caribe em Ciências da Saúde)	Endometriosis [DeCS] AND Parede [DeCS] AND Abdominal	4 case reports 4 case series 4 narrative reviews
SciELO (Scientific Electronic Library Online)	Endometriose [DeCs] AND Cesarea [DeCS] AND Complicações pós-operatórias [DeCS]	1 case series

MeSH = Medical Subjects Headings; DeCS = Descritores em Ciências da Saúde.

## RESULTS

**Figure 3.**
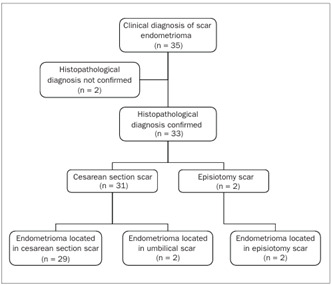
Diagnosing of scar endometrioma following cesarean section and vaginal delivery.

Operations to remove 35 scar nodules that had previously been diagnosed as endometrioma were performed between August 2001 and December 2007 (Figure 3). Two of these cases were excluded from this study: in these, the anatomopathological diagnosis was dermal fibrosis and surgical string granuloma. No other case was diagnosed during that period through pathological anatomy. The mean age of the patients at the time of the diagnosis was 30.1 ± 5.0 years, with a range from 18 to 41 years.

All of the patients described here had undergone an abdominal surgery event: either cesarean section or vaginal enlargement surgery (episiotomy). Of these, 31 (93.9%) were cesarean sections, and two (6.0%) were vaginal deliveries with episiotomy. The locations of the lesions were as follows: 29 (87.9%) under the cesarean section scar; two (6.0%) in the episiotomy scar, of which one was associated with the perianal area; and two (6.0%) in the umbilical scar, both associated with cesarean scars.

The total number of deliveries performed in the HMMEVNC over the study period was 29,135, and the total estimated incidence of scar endometrioma was thus 0.11%. Among the patients who underwent cesarean section, the incidence was 31 cases out of 10,533 deliveries (0.29%), and among those with vaginal deliveries, the incidence was two cases out of 18,602 deliveries (0.01%). By comparing these incidence rates after statistical calculations, we can conclude that cesarean section delivery implied a relative risk of 27.37 (P < 0.000001) for the occurrence of surgical scar endometriosis.

The mean number of pregnancies among the patients studied was two (standard deviation, SD: ± 1.43), with a range from one to seven pregnancies. Twenty-two of the patients (66.7%) had had one or two pregnancies (11 each). In relation to delivery type, most of the patients with scar endometrioma (69.7%), had only had one previous cesarean section performed (mode = 23). The two patients with episiotomy scar endometrioma had only had one previous vaginal delivery.

The main complaint was cyclical pain in the tumoral area, relating to the menstrual period, and this was found in 22 patients (66.7%). Localized pain in the scar area, without any relationship with the menstrual period, was reported by seven patients (21.2%). One patient (3.0%) had pelvic pain and another one (3.0%) had abdominal pain, which was cyclical in both cases but without any specific location. Three patients (9.0%) had complaints of painless nodules under the scar. There was only one case (3.0%) of a patient without any complaints and, in this case, the scar nodule was found through physical examination. In 31 patients (90.9%), the tumor was palpable under the previous scar, on physical examination. Among the non-palpable cases, two nodules (66.7%) were located under the cesarean section scar and one (33.3%) under the umbilical scar. Only one patient reported bleeding through the umbilical scar, and the tumor was located in that area.

The mean duration of the symptoms, reported at the first visit, was 30.5 months (± 25.1), with a range from 0 months (asymptomatic patient) to 8 years. The mean asymptomatic window period, defined as the time interval between the previous surgery and the onset of the symptoms, was 46.7 months (± 27.6), with mode and median equal to 48. In the episiotomy scar endometrioma cases (n = 2), the asymptomatic window was greater than 96 months.

The preoperative diagnosis was correct in 94.2% of the cases initially evaluated (n = 35), while bearing in mind that two cases were excluded because they were found not to consist of scar endometrioma, through histopathological evaluation.

All of the patients were treated surgically, with removal of the endometrioma with a safety margin, with the aiming of achieving a cure and avoiding locoregional recurrence. There were two cases of recurrence (6.0%) after the surgical treatment, both in umbilical scars, corresponding to 100% of the cases at that site. The operations were performed once again, with therapeutic success up to now, and these patients were not included in the study again. In 25 cases of endometriomas with previous cesarean section scar (80.6%), the lesion was supra-aponeurotic, in subcutaneous cellular tissue. The other ones (n = 6) compromised aponeurosis, muscle and/or peritoneum tissue, and were defined as subaponeurotic. All subaponeurotic cases were palpable. One case of cesarean section scar needed a propylene surgical screen for reconstruction of the aponeurosis, because of the large volume of tissue surgically removed.

## DISCUSSION

The longest study on scar endometriosis published so far described 72 cases that were evaluated over a 25-year period.^6^ The current study evaluated the frequency of scar endometrioma over a six-year period, with 33 cases, which is a proportionally representative number and, when compared with larger studies in terms of absolute numbers, it is highly relevant.^6^^,^^16^^,^^17^^,^^18^^,^^19^^,^^20^ Furthermore, all the cases described in the present study underwent surgery performed by the same team, which means that the team acquired great experience in handling this disease. Even though there have recently been studies presenting larger numbers of cases, better understanding should be reached through profiling the disease, searching for more efficient actions for prevention, diagnosis and therapeutics, and publishing such information in the medical media.

The research design was retrospective, through inclusion of cases with a definitive diagnosis of scar endometriosis. The histopathological criterion used is corroborated by the literature, and the slides were evaluated within the same service. The methodological limitation caused by the low frequency of the disease makes it impossible to perform well-controlled clinical trials, even in reference services dedicated to women’s health.

The incidence of endometriosis in the current study was estimated from the number of cases diagnosed in relation to the number of deliveries performed over the same period at the HMMEVNC. This is an estimate, and not an absolute value for the incidence, given that it is possible that some patients with this disease may have sought another place for treatment. On the other hand, all the patients studied had received delivery care at our service. The few studies that have described the incidence of endometriosis following delivery evaluated it in their services in the same way.^2^^,^^6^^,^^10^^,^^17^^,^^21^

A small number of studies have estimated the incidence of scar endometrioma following vaginal delivery alone.^6^ The rate relating to vaginal delivery in the present study was much lower (0.01%) than what had previously been reported, which had ranged from 0.06 to 0.7%. However, methodological factors compromised this specific incidence, probably because of lack of patient follow-up over the years after the delivery or different techniques used in general hospitals and maternity hospitals for performing episiotomies. With regard to the observed incidence of cesarean section scar endometrioma (0.29%), the present data corroborate the data in the literature reviewed, which consists of a greater number of studies available (compared with the number on vaginal delivery), although the data may vary due to social, cultural and legal factors. The difference in incidence between vaginal delivery and cesarean section can be explained by the greater manipulation during cesarean section, with exposure of the decidua to the subcutaneous tissue. The variation in incidence between studies, regarding the same procedure, might also relate to the various types of intra-surgical care and the care procedures during delivery.

Both scar endometrioma and pelvic endometriosis affect young women of reproductive age and, most frequently, multiparae between 25 and 35 years old.^20^ The average age of the patients in the current study was approximately 30 years and was in line with the age described in most other studies. The present study did not find any correlation between the number of pregnancies and parity and the risk of acquiring the disease. Thus, the number of successive cesarean sections did not seem to increase the risk, among the 23 patients (69.7%) in this study who had only had one previous cesarean section.

The most evident risk factor for the presence of endometriosis in scar tissue is a previous history of obstetric surgical procedures, especially cesarean sections, and hundreds of cases have been reported. The results from the current study corroborate this fact, since 100% of the patients had obstetric histories involving surgical procedures. A report published in 2007, with 117 cases, concluded that early hysterotomy (before the 22^nd^ week of pregnancy), alcohol consumption and increased menstrual flow are important risk factors.^18^ In evaluating the obstetric history of 81 patients with endometrioma, Wicherek et al. stated that performing cesarean sections without the presence of labor conditions more than doubles the risk in relation to situations in which cervical ripening and uterine contractions are present.^16^ The use of laparoscopic examinations and biopsy puncture has been correlated with umbilical scar endometriomas.^15^^,^^22^^,^^23^^,^^24^ Other rarer general surgical procedures, such as congenital inguinal hernia, might lead to endometrioma at the incision site, even mimicking hernia recurrence.^4^^,^^25^

In 1958, Ridley and Edward performed a successful experimental study on humans in which they injected endometrial tissue into the abdominal wall in order to corroborate the iatrogenic implant theory.^25^ Since then, this has been the main theory accepted by several authors in relation to the genesis of this disease.^11^^,^^12^^,^^26^ The result from the current study corroborates the theory of iatrogenic cell transportation. On the other hand, this theory alone is not enough to completely explain the physiopathology, given the low incidence of this disease and the reports on skin endometriosis without previous surgery, or scar endometriosis without opening the peritoneal or uterine cavities.^4^^,^^24^ Such events may be better explained by other theories or by combining all of them: coelomic metaplasia theory, lymphatic and/or hematogenic dissemination theory and cell immunity change theory. Moreover, appropriate estrogenic stimulation is needed in order to support all of these events, which explains why only women with functional ovaries are affected by this disease.

Currently, pregnancy is believed to provide immune tolerance to fetal antigens, and this inherent survival mechanism seems to be involved in the development of the endometrioma, consequently decreasing the cell immunity at locations where decidual cells are present. Thus, labor onset with cervical ripening and regular contractions would be a marker for the end of immune tolerance, because in the absence of this condition (elective cesarean section), labor seems to be a factor related to the disease.^16^ However, the ability of ectopic endometrial cells to resist cell apoptosis allows them to survive in the surgical scar. A recent study concluded that the expression of metallothionein through endometrial cells and receptor-binding cancer antigen through SiSo cells (RCAS1), i.e. the membrane antigens that control cytotoxic activity, might cause the scar cells in cesarean sections to persist.^27^

Ovarian hormonal action on ectopic endometrial cells (stromal and glandular cells) during the menstrual period causes slight bleeding at the scar location, with an inflammatory reaction and subsequent tissue repair.^8^ Thus, as each menstrual cycle goes by, the lesion increases in volume and behaves like an invasive tissue, which can be seen in histopathological examinations. Such invasion might compromise the skin, subcutaneous cellular tissue, muscles, aponeurosis and peritoneum. At certain points during the cycle, areas of focal hemorrhage can be identified, along with areas of active chronic endometriosis with fibrosis and cellular infiltration that is rich in macrophages and histiocytes loaded with hemosiderin pigments. However, that study^8^ did not consider these elements essential for the histopathological diagnosis, given that they might be missing.

Preoperative diagnoses of surgical scar endometrioma are often wrong, especially those made by general surgeons. This has led to the publication of certain papers in the literature, and this diagnostic difficulty has even been considered to be a dilemma among these papers.^5^^,^^13^^,^^22^^,^^23^^,^^28^^,^^29^^,^^30^ The nonspecific nature of the clinical presentation of endometrioma, along with the possible differential diagnoses, such as string granuloma, incisional hernia, hematoma, abscess, cysts and lipoma, are responsible for this diagnostic trap.^7^

The most evident clinical manifestation is a painful subcutaneous nodule, of chronic cyclical nature matching the menstrual period, with location in a surgical scar area. Twenty patients (60.6%) in the current study presented classic symptoms that were concordant with other reports published.^15^^,^^30^^,^^31^ Some authors consider such manifestations to be almost pathognomonic.^22^^,^^32^ The cyclical nature of the complaint is an important factor that predicts the disease and was present in 66.7% of the patients in the current study, despite what some authors claim.^6^^,^^10^^,^^28^ When the complaint is not cyclical, clinical diagnosis is impaired.^33^ Signs and symptoms may occur singly, which also impairs accurate diagnosis. There have been reports on acute manifestations of the symptoms (acute abdomen), which require emergency treatment.^29^^,^^34^ Other clinical forms, such as scar bleeding, pus and abdominal pain have been found in superficial lesions or umbilical scars.^20^^,^^25^

Physical examination is essential for an accurate diagnosis. Hard nodules are usually found by palpation of subcutaneous cellular tissue, below the scar and in any part of it. Depending on the thickness of the fat of the panniculus adiposus, the size of the nodule and the phase of the cycle, palpation might show a hard endometrioma. However, the length of the lesion does not seem to have any influence, since 100% (n = 6) of the subaponeurotic endometriomas were palpable in the current study.

An association between scar endometriosis and pelvic endometriosis is found in one quarter of the cases, thereby making it possible to provide greater applicability for pharmacological treatment.^12^^,^^19^^,.^^35^ On the other hand, routine pelvic cavity investigation by means of laparoscopy is not recommended.^12^

Malignization is a rare event, occurring in 0.3-1% of scar endometriomas. Clear cell carcinoma is the most common histological subtype, and the 20-month survival rate is only 57%.^36^ Frequent recurrence might indicate malignant degeneration of the tumor. Even though this is a rare disease, there is a recommendation for long clinical follow-up on all cases, because the interval between the onset of scar endometriosis and its malignant transformation might vary from a few months to more than 40 years.^37^

Auxiliary propaedeutics might be necessary at times to explain the diagnosis and make a better plan for the surgical treatment to be performed. This is justified only in cases of large lesions, doubtful diagnosis and atypical clinical manifestations. In some cases, thin needle puncture guided by ultrasound, with cytological analysis, helps to confirm the diagnosis. However, its use is still controversial because of the risk of causing new implants at the puncture sites or perforating a hollow organ, in the case of an incarcerated hernia that simulates endometrioma.^8^^,^^35^ Likewise, laparoscopic examinations in cases relating to pelvic endometriosis are not recommended for the same reasons.

**Figure 4.**
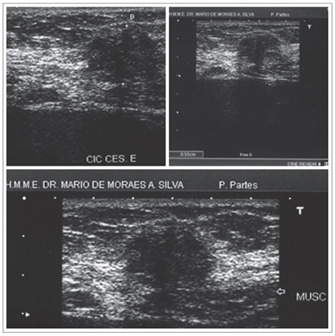
Ultrasound scan on a case of endometrioma in the abdominal wall (cesarean section scar). Details show hypoechoic image with posterior acoustic shadow.

Ultrasound is a good investigational method for tumoral masses, given its practicality and low cost. Abdominal wall ultrasound shows a solid, hypoechogenic and vascularized image, with the possibility of cyst components of mixed echogenicity. Although ultrasound is nonspecific, findings close to cesarean section scars strongly suggest a diagnosis of endometrioma ^8^^,^^11^^,^^21^ (Figure 4).

Because of the highly specific resolution of magnetic resonance imaging (MRI), this technique makes it possible to identify smaller lesions and distinguish signs of organized hemorrhage within endometriomas, thereby suggesting this diagnosis.^8^^,^^38^ Moreover, MRI has better performance than computed tomography (CT) scans in relation to outlining the subcutaneous, muscle and aponeurotic tissue layers.^38^ CT scans show heterogenous growth with variable density in cross-sections, corresponding to abdominal wall lesions.^8^

Imaging examinations are usually unnecessary from a practical point of view, considering that most cases are diagnosed relatively successfully, simply by means of a good initial questionnaire and physical examination. In our case series, despite all of the advanced propaedeutic elements, the diagnostic hypothesis was correct in 94.3% of the cases simply through anamnesis and physical examination, and occasionally, ultrasound. It also needs to be borne in mind that CT and MRI scans are not readily available in the Brazilian public health system, because of their high cost. Therefore, the use of these examinations is restricted to surgical planning, such as in cases of larger lesions, or in order to establish differential diagnoses, when there is some doubt. In the current study, no attempt was made to evaluate the size of the lesion, because auxiliary diagnostic evaluations using ultrasound were not performed in all cases and, when performed macroscopically, such evaluations can be overruled by the surgical safety margin. The literature reviewed showed that the average lesion size ranged from 2.3 to 3.2 cm, while the largest lesion measured 14 cm.^2^^,^^6^^,^^12^^,^^17^^,^^28^

**Figure 5.**
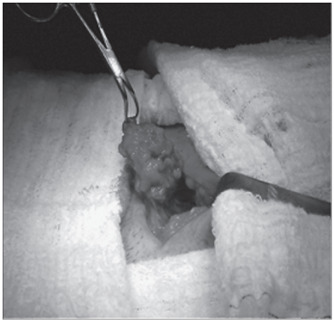
Scar endometrioma in cesarean section scar: dissection and excision of supra-aponeurotic lesion.

In the same way as in the literature reviewed, we recommend that surgical removal of the nodule should be performed with a safety margin, as the preferred treatment. In addition to decreasing the chances of local relapse, this procedure enables safe treatment when the tumor happens to be malignant. The excision may be technically difficult, depending on the depth and the size of the mass, and the surgical mass excised may need to be enlarged if the lesion extends to deeper tissues, such as aponeurosis, muscle or, more rarely, peritoneum (Figure 5). Among the cases in the present study, only one case required a propylene surgical screen, in order to repair the aponeurosis because of wide dissection of that tissue. Another possible surgical tactic is to rotate the aponeurotic muscle flap.^2^ The relapse cases in the current study (n = 2) were only treated successfully with new surgery. However, in the literature, associated hormone therapy has been suggested for such cases.^2^^,^^14^

Some authors have recommended specific techniques depending on where the endometrioma is located. Barisic et al. advocated wide excision of the nodule with primary sphincteroplasty, in order to achieve good healing with preservation of fecal continence in cases of scar endometrioma due to episiotomy involving the anal sphincter.^39^ Kokuba et al. found that a technique involving two semicircular defatted flaps was efficient for creating a neo-umbilicus after removing umbilical scar endometrioma.^40^ The curative surgery should preferentially be performed some days before the menstrual period, thus avoiding an inflammatory reaction and making tissue removal easier.^8^

Although the treatment of choice is surgical, several authors have advocated the use of hormone therapy in special cases, with the main aim of decreasing the size of the tumor mass and facilitate a further surgical procedure. Other possible indications for this are in situations of relapse or in relation to pelvic endometriosis.^2^^,^^14^^,^^15^^,^^34^^,^^38^ Victory et al. stated that the use of drug therapy might result in long-term control over the symptoms, with minimal risk of malignization.^22^ Generic forms of gonadotropin-releasing hormone (GnRH), danazol and progesterone at the same doses have been approved for treating pelvic endometriosis and, consequently, such patients are subject to the side effects of these drugs, such as amenorrhea.^15^

Taking the main physiopathogenesis of scar endometrioma as the main theory, several measures have been proposed for prevention of iatrogenic implantation in the endometrium, but without any evident scientific corroboration. Use of good surgical techniques and intra-surgical tests are deemed elementary precautions in this respect.^2^^,^^8^^,^^20^^,^^35^ Failure to close the parietal and visceral peritoneum in the cesarean section may be related to greater rates of scar endometrioma.^17^ It is recommended not to use the same surgical material and the same instruments as used in hysterorraphy, when suturing other abdominal wall layers.^2^^,^^8^ Some authors have reported ongoing use of high doses of progesterone in order to decrease the occurrence of endometriosis at the surgical site, during the first six months after hysterotomy.^2^ Other authors have recommended washing the abdominal wall as a prophylactic measure, using irrigation with a salt solution before closing the wall.^8^^,^^41^ To sum up, although there are no well-controlled published clinical trials that can strengthen this topic through better evidence, we agree that adopting sensible care during the surgical procedures is highly recommendable.

## CONCLUSION

The current study showed endometrioma incidence rates similar to those found in the literature reviewed, thus leading to the conclusion that this uncommon disease is, however, not at all rare. Observation of the clinical history of the patients studied helped to determine the genesis of scar endometrioma, thereby corroborating the theory of iatrogenic transportation. The most relevant clinical data was the cyclical characteristic of pain that coincides with the menstrual period (66.7%). Patients who underwent cesarean section presented greater risk of developing endometrioma in the scar location, compared with patients who underwent vaginal delivery (relative risk, RR = 27.37 and P < 0.01). The diagnosis was correct in almost all the cases, and was achieved simply by using ultrasound in conjunction with the patient’s clinical history (94.3%). The treatment of choice was wide excision of the lesion with a safety margin. Up-to-date review of the literature contributed towards accurate diagnosis, thus avoiding inappropriate procedures and unnecessary expenditure in caring for the disease.

Furthermore, dissemination of this information outside of the field of obstetrics and gynecology may be useful for guiding other physicians regarding the correct therapeutic approach to adopt, thus preventing recurrence and malignant disease. There is a need for new studies in order to determine which prophylactic measures are most efficient. Lastly, understanding scar endometrioma as a disease originating essentially during the period of delivery care may lead to rethinking of the best practices and care relating to this period.
